# Emphysematous Cystitis and Urinary Retention in a Male Patient With Diabetes Mellitus Type 2 Treated With Empagliflozin

**DOI:** 10.1016/j.aace.2022.04.002

**Published:** 2022-04-08

**Authors:** Gina M. Brock, Sarah M. Lane, Theodore S. Roosevelt

**Affiliations:** 1 Idaho College of Osteopathic Medicine, Meridian, Idaho; 2 Idaho Diabetes-Endocrine Associates, Boise, Idaho

**Keywords:** emphysematous cystitis, empagliflozin, SGLT2 inhibitors, urinary tract infection, diabetes mellitus type 2, CT, computed tomography, EC, emphysematous cystitis, ER, extended-release, FG, Fournier gangrene, GU, genitourinary, HbA1c, hemoglobin A1c, LUT, lower urinary tract, PVR, postvoid residual, SGLT2i, sodium-glucose cotransporter 2 inhibitor, T2DM, type 2 diabetes mellitus, US, ultrasound, UTI, urinary tract infection

## Abstract

**Objective:**

Emphysematous cystitis (EC) is a rare urinary tract infection (UTI) typically associated with severe diabetes in older women. We present a unique case of this gas-forming infection in a man with type 2 diabetes mellitus (T2DM) treated with empagliflozin. To the best of our knowledge, this is the first case report of EC associated with the use of a sodium-glucose cotransporter 2 inhibitor (SGLT2i).

**Case Report:**

A 62-year-old man with T2DM treated with an SGLT2i developed EC. His moderately controlled T2DM was treated for over 20 years with metformin, saxagliptin/metformin, and pioglitazone to which empagliflozin was added due to his consistently elevated hemoglobin A1c level, slightly reduced estimated glomerular filtration rate, and proteinuria. Four months after initiation of the SGLT2i, he reported lower urinary tract symptoms and was found to have EC radiographically. His urine cultures were positive for *Klebsiella pneumonia* and was found to have asymptomatic urinary retention. He was treated conservatively, and his outcome was favorable.

**Discussion:**

EC is commonly seen in patients with diabetes mellitus, and symptoms range from asymptomatic to severe sepsis. Most urine cultures grow *Escherichia coli* and *K. pneumonia*. The association of increased UTIs in susceptible patients with T2DM with the use of SGLT2i is yet to be determined. Most cases of EC are diagnosed radiographically and treated conservatively, although some cases require surgical intervention.

**Conclusion:**

Initially, our patient was considered a good candidate for treatment with an SGLT2i. The subsequent development of EC precluded its further use. The role of SGLT2i in patients with T2DM susceptible to UTI is controversial.

**Highlights**

• Emphysematous cystitis (EC) is a rare, but severe gas-forming urinary tract infection commonly seen in patients with diabetes mellitus with and without comorbid conditions predisposes to bladder infections.
• The use of sodium-glucose cotransporter 2 inhibitors (SGLT2i) in type 2 diabetes mellitus in patients susceptible to urinary tract infection is still being determined.
• Sodium-glucose cotransporter 2 inhibitors use in patients with diabetes mellitus may increase risk for EC.
• EC is commonly diagnosed radiographically and usually treated conservatively.

**Clinical Relevance**

The US Food and Drug Administration has encouraged use of SGLT2i for patients with reduced ejection fraction in heart failure with and without type 2 diabetes mellitus. Practitioners should be aware of potential risk of EC, a rare but severe infection, should they prescribe this medication. There is evidence of increase of Fournier’s gangrene, another severe gas-forming infection, with use of SGLT2i in diabetes mellitus. The risk-benefit needs to be weighed in susceptible patients.

## Introduction

Emphysematous cystitis (EC) is a rare form of complicated urinary tract infection (UTI) that presents with air within the bladder wall and lumen. As of 2019, there were approximately 240 reported cases of EC.^1^, ^2^, ^3^ EC has a higher incidence among older (mean age, 66 years), severely diabetic women and is more commonly associated with urologic conditions that promote urinary stasis and recurrent UTIs.^1^ The indications for sodium-glucose cotransporter 2 inhibitors (SGLT2i) in diabetic patients with an increased risk of UTIs are yet to be determined.^4^ After reviewing the appropriate literature, we found that this is the only case report known to the authors associated with the use of an SGLT2i.

## Case Report

A 62-year-old male patient with type 2 diabetes mellitus (T2DM) and hypertension had his condition moderately controlled with metformin extended-release (ER) 500 mg daily, saxagliptin/metformin ER 5 mg/1000 mg daily, and pioglitazone 45 mg daily. In early 2014, his hemoglobin A1c (HbA1c) level was 6.3% (45 mmol/L). It increased to 6.8% (51 mmol/L) in late October 2014. He was employed as a truck driver, which made optimizing diet and exercise regimens difficult. Therefore, empagliflozin 10 mg daily was added to his medication regimen.

**Fig. 1:**
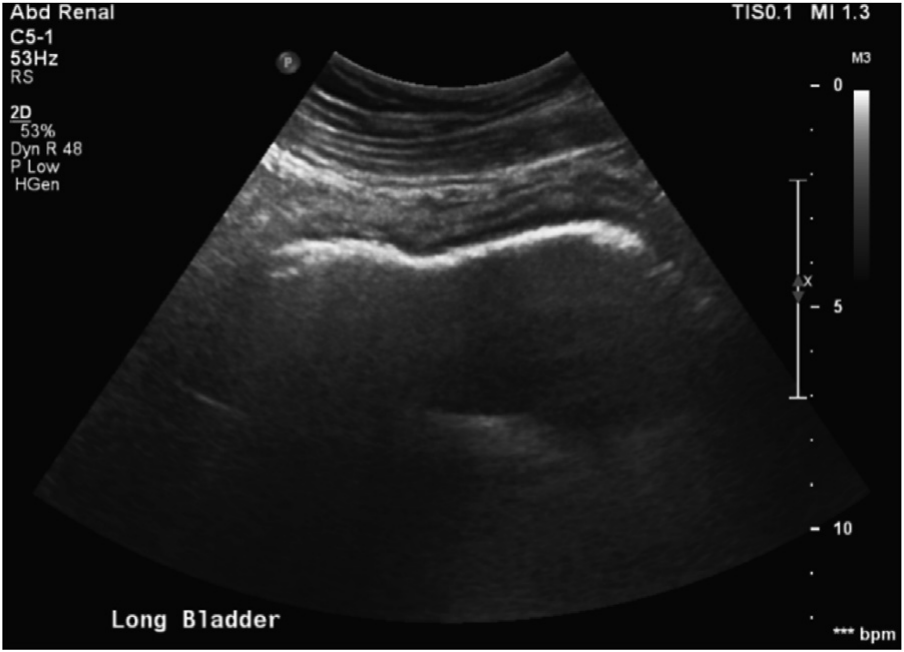
Bladder ultrasound demonstrating echogenic gas in the bladder wall.

On February 13, 2015, he presented to the clinic with 8 hours of painless gross hematuria, urinary frequency, and urgency. He complained of mild lower abdominal pain and denied dysuria, fever, chills, nausea, vomiting, and groin pain. On examination, he had mild suprapubic abdominal tenderness but appeared otherwise in good health. The patient was sent for urinalysis, complete blood count, renal panel, and an urgent genitourinary (GU) ultrasound (US). The US revealed EC, which demonstrated echogenic gas within the bladder wall as shown in Figure 1.

On admission, he was conversant and resting comfortably. The patient denied any history of previous UTI, gross hematuria, urinary retention, or nephrolithiasis. He denied feeling ill the day before or the day of the presentation. There was no history of GU surgery or instrumentation. His cardiovascular and pulmonary examinations were unremarkable, with no extremity edema. The abdominal examination was unremarkable without costovertebral angle tenderness or gross abnormalities seen on his GU examination. Admission vital signs and laboratory data are displayed in Table 1.

**Table 1:** Patient Admission Vital Signs and Laboratory Data

Vital signs	Serum laboratory results	Urinalysis
Blood pressure: 153/75 mm Hg	White blood cells: 10 × 10^3^/μL	Specific gravity: 1.025
Pulse rate: 85 beats/min	Neutrophils: 74%	Occult blood: 3+
Respiratory rate: 16 breaths/min	Hemoglobin: 13.8 g/dL	Leukocyte esterase: 3+
Oxygen saturation: 95% SpO2 RA	Platelet count: 226 × 10^3^/μL	Protein: 3+
Temperature: 36.3°C	Blood urea nitrogen: 14 mg/dL	Glucose: 3+
BMI: 29.5 kg/m^2^	Creatinine: 0.78 mg/dL	Nitrites: positive
	Total CO_2_: 23 mmol/L	Red blood cells: >50/high-power field
	eGFR: >60 mL/min/1.73 m^2^	White blood cells: 2-5/high-power field
	Glucose: 132 mg/dL	
	HbA1c: 7.6% (60 mmol/L)	
	Transaminases: unremarkable	

Abbreviations: BMI = body mass index; eGFR = estimated glomerular filtration rate; HbA1c = hemoglobin A1c; SpO2 RA = oxygen saturation on room air.

Computed tomography (CT) of the abdomen and pelvis with intravenous contrast noted intramural gas in the bladder. There was no urinary outlet obstruction or fistula formation noted on US or CT. Soon after, the patient complained of worsening bladder fullness. His postvoid residual (PVR) demonstrated volumes of >200 mL suspicious for inadequate emptying of the bladder. A Foley catheter was placed, and the patient was started on tamsulosin 0.4 mg daily. His diabetes was managed with insulin, and all oral agents were discontinued. He was started on intravenous ceftriaxone 1 g daily. His urine culture grew >100 000 colonies/mL of *Klebsiella pneumonia*, susceptible to fluoroquinolones and ceftriaxone. His blood cultures were negative, and ceftriaxone was discontinued, followed by initiation of oral ciprofloxacin 750 mg twice daily.

During his 2-day hospital course, the patient remained stable and did not develop a systemic illness. His admission was uneventful, apart from persistent elevated PVR volumes with an attempt to remove the Foley catheter on discharge. Except for empagliflozin, the patient restarted his previous hypoglycemic medications. He continued tamsulosin in addition to a Foley catheter. The Foley catheter was removed 6 days later after a successful voiding trial. He continued to have elevated but stable PVR volumes (>200 mL) on follow-up despite treatment with tamsulosin. His urinalysis was normalized, and near-total resolution of EC was noted on CT 20 days after discharge. After returning to work, he was lost to follow-up and did not undergo cystoscopy recommended by the urologist after discharge. He returned to the urology clinic 8 months later with complaints of malodorous urine. He was found to have an HbA1c level of 7.2% (55 mmol/L) and a body mass index of 32 kg/m^2^. He was started on ciprofloxacin 500 mg twice daily for 10 days with resolution of his symptoms but never returned to the urologist for follow-up evaluation of causes of his urinary retention.

In 2017, at follow-up in the endocrinology clinic, the patient reported that he retired and adopted a no-white food diet, gardening, and walking 2 to 3 mi, 4 times per week. In early 2020, he discontinued saxagliptin/metformin ER because of improving blood sugar levels, but he reported continued use of tamsulosin without reoccurrence of his urinary symptoms. In August 2020, his HbA1c level was 6.5% (48 mmol/L), and his body mass index was 26 kg/m^2^.

## Discussion

EC is a rare gas-forming infection of the bladder wall. The strongest risk factor is diabetes mellitus (70%).^2^ Thomas et al^1^ found that 64% of EC cases occurred in women and 67% were diabetic. Other risk factors of EC include neurogenic bladder, recurrent UTIs, bladder outlet obstruction, and instrumentation of the bladder.^1^^,^^2^ It is the most common of the emphysematous infections of the abdomen and pelvis with a 7% mortality rate. Infections of the gallbladder, stomach, pancreas, and GU system, including Fournier gangrene (FG), are associated with 15% to 80% mortality.^1^^,^^2^^,^^5^ Pneumaturia is a specific and rare symptom of EC, and the predominant presentation is nonspecific and includes symptoms of abdominal pain, hematuria, and lower urinary tract (LUT) symptoms as seen in this patient.^1^, ^2^, ^3^ Some patients experienced fever (30%-50%), bacteremia (50%), and elevated white blood cell counts (14 × 10^3^/μL). There are various presentations of the disease that range from asymptomatic infection to severe sepsis.^1^^,^^2^^,^^5^ Most urine cultures grow *Escherichia coli* (58%) and *K. pneumonia* (21%).^1^^,^^2^

EC is commonly diagnosed radiographically. Emphysematous infections of the abdomen and pelvis are separate and distinct; however, they share common features. Several patients have either poorly controlled diabetes mellitus, vascular disease, tissue infarction, trauma, or high tissue glucose levels, thought to be the substrate for gas-producing organisms.^1^^,^^2^^,^^5^ CT is the preferred imaging modality to help delineate the extent and location of the disease, fistula formation, intra-abdominal abscess, and neoplasm.^5^

Glucose reabsorption may increase by as much as 20% in patients with T2DM from altered expression and the activity of SGLT2 receptors.^6^ SGLT2i reduces renal absorption of filtered glucose and lowers the renal tubular threshold for glucosuria, enhancing glucose excretion.^6^ In 2015, the U.S. Food and Drug Administration issued a warning for SGLT2i to include an increased risk of severe UTI infections.^7^ A large population-based cohort study, identifying over 235 000 patients with T2DM divided into 2 cohort arms, found no increased risk of severe UTI with SGLT2i compared with that of dipeptidyl peptidase-4 inhibitors or glucagon-like peptide-1 receptor agonists.^7^ Other studies reported that SGLT2i caused mild to moderate UTIs.^4^^,^^7^ However, a 2016 meta-analysis of 77 randomized control trials found no significant risk of UTIs with SGLT2i.^8^ Interestingly, this patient was on a dipeptidyl peptidase-4 inhibitor and SGLT2i concurrently, but only the SGLT2i was removed from his medication regimen probably because of this new Food and Drug Administration warning. It is plausible to consider that SGLT2i was a catalyst that initiated the EC infection, despite his other comorbid risk factors, noncompliance, and return of LUT symptoms after cessation of empagliflozin. He initiated the SGLT2i 4 months before EC. Before then, he never experienced or complained of LUT symptoms to his providers for over 20 years. However, the patient refused further evaluation to elucidate the cause of his urinary retention fully. Diabetic nephropathy may also have contributed to his presentation.

SGLT2i are associated with an increased risk of FG in T2DM.^8^^,^^9^ Between 2004 and 2019, empagliflozin was found to be associated with the highest number of FG reports (232/542) in the SGLT2i drug class with the highest mortality, followed by canagliflozin and dapagliflozin, but the percentage of cases among all patients receiving SGLT2i is unknown.^9^ There was a significant increase (407) of FG cases identified with the use of SGLT2i in 2014 to 2019, suggesting increased SGLT2i use in T2DM.^9^

Despite concerns, the positive effects of empagliflozin and other SGLT2i cannot be ignored. Empagliflozin was the first in its class to demonstrate a reduction in mortality by reducing cardiovascular events in patients with T2DM.^6^ There was also a reduction in body weight and blood pressure and prevention of heart and renal failure with a lowered risk of hypoglycemia, all while reducing insulin doses and HbA1c levels (1%-1.5%).^6^ Initially, this patient was an excellent candidate to benefit from SGLT2i because of his consistently elevated HbA1c levels, slightly reduced estimated glomerular filtration rate, and proteinuria. However, this patient developed a severe gas-forming bladder infection after the initiation of an SGLT2i in the setting of undiagnosed mild urinary retention with relatively controlled T2DM. A similar report was published in a male diabetic patient with a PVR volume of 180 mL who developed a UTI after initiation of an SGLT2i.^10^ This suggests that practitioners should consider screening patients for obstructive symptoms before the start of SGLT2i to assess the risk of developing severe UTIs, such as EC.

The majority (90%) of patients with EC are managed successfully with fluid resuscitation, targeted antibiotic therapy, augmentation of risk factors, and bladder rest via Foley catheter drainage.^1^^,^^2^^,^^11^ Hyperbaric treatment has been used successfully.^12^ Only 10% of patients with EC required surgical intervention.^1^

## Conclusion

We reported a case of EC in a man with T2DM after initiation of empagliflozin with subsequent asymptomatic urinary retention. Treatment for EC was conservative. We provided a literature review of EC and discussed the benefits and adverse drug effects of SGLT2i in T2DM. The outcome of EC was favorable in this patient.
